# P-1012. Looking Back to Move Forward: Insights into Current Coccidiomycosis Therapeutic Pathways

**DOI:** 10.1093/ofid/ofae631.1202

**Published:** 2025-01-29

**Authors:** Brent Edwards, Rebecca A Ripperton, Daniel B Chastain, George R Thompson, Andrés F Henao Martínez

**Affiliations:** University of Colorado Denver, Department of Medicine, Division of Infectious Diseases, Aurora, Colorado; University of Colorado Denver, Department of Medicine, Division of Infectious Diseases, Aurora, Colorado; University of Georgia College of Pharmacy, Albany, GA; University of California Davis Medical Center, Sacramento, CA; University of Colorado Anschutz Medical Campus, Aurora, Colorado

## Abstract

**Background:**

Coccidioidomycosis (cocci) poses therapeutic challenges due to the variability in disease presentation and severity, particularly for immunocompromised patients. Treatment approaches for cocci have not changed in decades. Fluconazole may remain ineffective as a first-line option in refractory cases or in more invasive disease.

We aim to identify a cohort of patients diagnosed with cocci and their treatment pathways using a "real world" international global health network database.

**Table 1.**
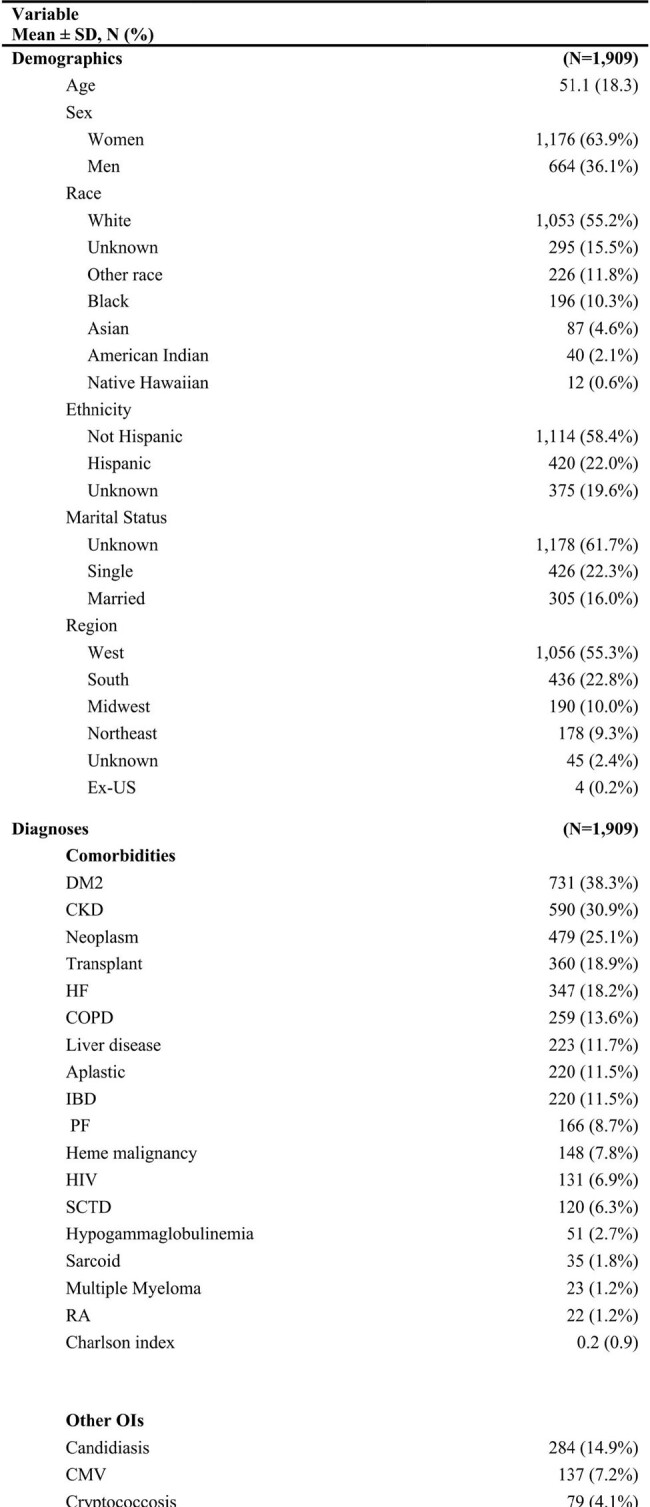
Characterization of patients undergoing treatment for coccidiomycosis including clinical characteristics and clinical outcomes.

**Methods:**

We conducted a multicenter retrospective study by querying TriNetX, a global research network, identifying patients with cocci by ICD-10-CM codes and linking to antifungal therapy. We investigated comorbidities diagnosed before infection and outcomes of interest after 1 year. Treatment pathways were assessed within 3 months post-diagnosis. A line of treatment was defined as receipt of the same medication within 3 days of cocci diagnosis, and was considered complete once absent from the patient’s record for 3 consecutive days. Graphs were designed with R Studio (3.6.0).

**Figure 1.**
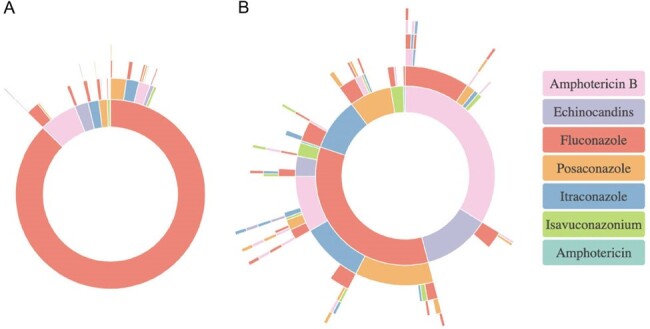
Treatment pathways in coccidioidomycosis Sunburst diagram of initial treatment choices for cocci. A. Treatment pathways for the entire patient cohort diagnosed with coccidioidomycosis B. A subset of the treatment pathway analysis that removes patients with fluconazole monotherapy to better visualize other treatments that were used. Each ring represents a line of treatment. The inner side of the ring is the initial treatment choice, while subsequent rings represent switches. Each switch was defined by taking the same medication for 3 days within coccidioidomycosis diagnosis followed by the receipt of a different medication for at least 3 consecutive days.

**Results:**

We captured 1909 patients diagnosed with cocci, of which 1581 (82.9%) had treatment pathway information. Most patients were white females around 51 years of age, presenting with pulmonary cocci (52%). Common comorbidities included T2DM (38.3%), CKD (30.9%), concurrent neoplasm (25.1%), and post-transplant-status (18.9%) (Table 1). Candidiasis was present in 14.9% of patients and CMV in 7.2%. Overall, 1-year mortality was 11%. Fluconazole was the most common initial therapy (87.4%) followed by amphotericin B (6.45%), echinocandins (2.34%), and itraconazole (1.83%). Of these, 11.2% of patients were switched to at least one new agent, with the most common switch being fluconazole to amB (1.6%) and amB to fluconazole (1.8%); 1.5% of patients were treated with ≥ 3 total antifungal agents (Fig 1).

**Conclusion:**

Fluconazole remains the preferred initial agent to treat cocci; only 12.6% of patients received an alternative agent. Most initial switches favored amB, raising concern for treatment failure with fluconazole. Unveiling clinical characteristics of those patients may reveal possible clinical predictors of failure or situations where amB may be preferred.

**Disclosures:**

**George R. Thompson, III, MD**, Astellas: Advisor/Consultant|Cidara: Advisor/Consultant|Cidara: Grant/Research Support|F2G: Advisor/Consultant|F2G: Grant/Research Support|Melinta: Advisor/Consultant|Melinta: Grant/Research Support|Mundipharma: Advisor/Consultant|Mundipharma: Grant/Research Support|Pfizer: Advisor/Consultant

